# Editorial: Food Bioactive Polysaccharides and Their Health Functions

**DOI:** 10.3389/fnut.2021.746255

**Published:** 2021-09-27

**Authors:** Tao Feng, Xingbin Yang, Qingjun Kong, Jun Lu

**Affiliations:** ^1^School of Perfume and Aroma Technology, Shanghai Institute of Technology, Shanghai, China; ^2^College of Food Engineering and Nutritional Science, Shaanxi Normal University, Xi'an, China; ^3^School of Public Health and Interdisciplinary Studies, Faculty of Health and Environmental Sciences, Auckland University of Technology, Auckland, New Zealand

**Keywords:** polysaccharide, health function, structure, conjugate, starch

Polysaccharides are a class of macromolecular compounds consisting of more than 10 identical or different monosaccharides linked by glycosidic bonds, which are widely found in living organisms. Among them, those polysaccharides that promote the health of the organism, control cell differentiation, regulate cell growth and aging, and participate in many physiological activities such as cell recognition, cell metabolism, embryonic development, viral infection, and immune response are called bioactive polysaccharides or biological response modifier (BRM). The chemical structure of active polysaccharides determines their biological activity, and the study of the conformational relationship of active polysaccharides can provide theoretical guidance for the purposive screening of their biological activity. With the characteristics of high safety, low toxic side effects, good efficacy and wide sources, active polysaccharides have received wide attention from scholars domestically and worldwide in recent years and has become one of the research hotspots in food science, medicine, and molecular biology.

Today, both food safety and food security affect our daily life greatly. This was our motivation to launch this Research Topic about bioactive polysaccharides in foods and their health functions. This Research Topic contains eight published papers, which very well represent the current knowledge in their respective fields.

For example, the first paper came from Li et al., titled “Relationships Between Cell Structure Alterations and Berry Abscission in Table Grapes.” In their paper, the authors established an abscission zone of grape berries characterized by multiple cell microstructural or ultrastructural alterations in the stalk-berry junction, berry brush, and pedicel tissue cells. They found that the detachment force of the grapes decreased and berry abscission increased over the mean time during the whole storage period. They also concluded that the “Nanyu” variety appeared to be best at delaying berry drop, whereas “Kyoho” and “Muscat Kyoho” appear to be very susceptible to berry drop. Their study will guide the grape-planting farmers to choose a good variety to improve berry abscission and keep the grape being ripe without dropping, thus increasing the harvesting ratio of the grape. The second paper dealt with gum arabic modulating hepatic and renal profile among rheumatoid arthritis sufferers. They chose 40 patients aged 18–70 to take gum arabic orally for 12 weeks as a single dose of 30 g. They finally concluded that GA presented hepatic and renal protective effects among RA patients. GA acted as an anti-inflammatory and antioxidant agent among end-stage hepatic and renal patients.

The third paper mainly discussed the evolution of fructans in Aguamiel during the plant production lifetime. They examined aguamiel composition in three agave plants during their productive lifetime (4–9 months). They finally found that the concentration of agave fructans and sucrose, especially fructan profile, change during the aguamiel production process. They even found some new fructo-oligosaccharides which can be synthesized by bacteria present in aguamiel. This study might play an important role in utilizing utilize aguamiel soon.

Researchers from China and the US cooperatively studied the impacts of environmental factors on the pasting properties of cassava flour mediated by its macronutrients. They finally reached a conclusion that the pasting temperatures of cassava flour had a significant direct correlation with growth temperature and were negatively correlated with altitude. They put forward a hypothesis that they might predict and control cassava flour quality and pasting properties via environmental condition alterations soon.

The fifth paper mainly studied the inhibitory effects of Hericium erinaceus beta-glucan (HEBG) on *in vitro* starch digestion. They firstly elucidated the fine structure of beta-glucan extracted from Hericium erinaceus, and then investigated the effects of the glucans on *in vitro* digestion of wheat starch by the Englyst method. Finally, they unraveled the triple helix structure in HEBG, which might play an important role in inhibiting starch digestion. Moreover, beta-1,3-glucans had stronger inhibitory effects than beta-1,6-glucans.

Several scientists jointly investigated fucoidan from Sporophyll of Undaria pinnatifida in Weihai, China. They mainly compared the chemical compositions and antioxidant activity of different molecular weight fractions. They used dialysis to get fucoidan fractions with molecular weight cutoffs of > 300 kDa and <10 kDa. Finally, they found that the primary antioxidant activity of the 10 kDa fraction was significantly higher than that of the 300 kDa fraction, fucoidan crude extract, and fucoidan standard, but the secondary antioxidant capacities of the 10 and 300 kDa fraction were similar, and both were higher than that of butylated hydroxy anisole (BHA). The ferric reducing antioxidant power was higher in the 300 k and fucoidan crude extract. These studies lay the foundation for a fucoidan industry in Weihai, China.

Chaudhari et al. from India reviewed curdlan therapeutic and industrial applications by considering recent patents. They firstly determined that curdlan is an exopolysaccharide composed of glucose linked with β-(1,3)-glycosidic bond. They then provided the latest information about the chemistry and biosynthesis of curdlan and its applications for making novel dairy products, functional foods, and nutraceuticals and details about the recent patents of curdlan and its derivatives.

Lv et al. from China contributed the final paper titled “structural features and digestibility of corn starch with different amylose content.” From their study, they found that waxy corn starch (WCS) and corn starch (CS) have the highest digestibility, while high amylose corn starch (HACS) has a higher content of resistant starch (RS). HACS 60, with the highest RS content, had a unique surface fractal structure, indicating that the dense structure is effective in maintaining the RS content. These conclusions made a fundamental study toward the development of more slowly digestible foods.

From the above summaries of the mentioned papers, it was seen that polysaccharides play essential roles for plants, animals, and human beings. However, eight papers can't represent all aspects related to food bioactive polysaccharides and their health functions. The following aspects might be considered as future foci:

1. Polysaccharides modulating the gut microbiota of human beings to control chronic diseases.
2. Polysaccharides changing food microstructure to control starch retrogradation, reduce ice crystals, and improve food mouthfeel.
3. Polysaccharides interacting with other macromolecules in food, such as protein or fatty acids, and generating different physicochemical and/or biological properties of these conjugates.
4. Polysaccharides interacting with each other thus forming food gel or a food colloidal system.
5. Polysaccharides acting as wall materials to encapsulate bioactive or flavor compounds.
6. Polysaccharides acting as Pickering agents to stabilize food flavor emulsions or food nutrients microemulsions.
7. Polysaccharides acting as fat replacers to substitute food lipids so as to prepare fat-reduced foods.
8. Polysaccharides acting as functional materials to prepare bioactive packaging, smart coating, and biodegradable materials.

## Author Contributions

TF: revising. XY and QK: writing and original idea. JL: organizing. All authors contributed to the article and approved the submitted version.

## Conflict of Interest

The authors declare that the research was conducted in the absence of any commercial or financial relationships that could be construed as a potential conflict of interest.

## Publisher's Note

All claims expressed in this article are solely those of the authors and do not necessarily represent those of their affiliated organizations, or those of the publisher, the editors and the reviewers. Any product that may be evaluated in this article, or claim that may be made by its manufacturer, is not guaranteed or endorsed by the publisher.

